# Rapid development of intestinal cell damage following severe trauma: a prospective observational cohort study

**DOI:** 10.1186/cc7910

**Published:** 2009-06-08

**Authors:** Jacco J de Haan, Tim Lubbers, Joep P Derikx, Borna Relja, Dirk Henrich, Jan-Willem Greve, Ingo Marzi, Wim A Buurman

**Affiliations:** 1 Department of Surgery, NUTRIM School for Nutrition, Toxicology and Metabolism, Maastricht University Medical Center, Universiteitssingel 50, 6229 ER, Maastricht, The Netherlands; 2 Department of Surgery, Orbis Medisch Centrum, Dr. H. van der Hoffplein 1, 6162 BG, Sittard-Geleen, The Netherlands; 3 Department of Trauma Surgery, J.W. Goethe University, Theodor-Stern-Kai 7, 60590, Frankfurt am Main, Germany; 4 Department of Surgery, Atrium Medisch Centrum, Henri Dunantstraat 5, 6419 PC, Heerlen, The Netherlands

## Abstract

**Introduction:**

Loss of intestinal integrity has been implicated as an important contributor to the development of excessive inflammation following severe trauma. Thus far, clinical data concerning the occurrence and significance of intestinal damage after trauma remain scarce. This study investigates whether early intestinal epithelial cell damage occurs in trauma patients and, if present, whether such cell injury is related to shock, injury severity and the subsequent inflammatory response.

**Methods:**

Prospective observational cohort study in 96 adult trauma patients. Upon arrival at the emergency room (ER) plasma levels of intestinal fatty acid binding protein (i-FABP), a specific marker for damage of differentiated enterocytes, were measured. Factors that potentially influence the development of intestinal cell damage after trauma were determined, including the presence of shock and the extent of abdominal trauma and general injury severity. Furthermore, early plasma levels of i-FABP were related to inflammatory markers interleukin-6 (IL-6), procalcitonin (PCT) and C-reactive protein (CRP).

**Results:**

Upon arrival at the ER, plasma i-FABP levels were increased compared with healthy volunteers, especially in the presence of shock (*P *< 0.01). The elevation of i-FABP was related to the extent of abdominal trauma as well as general injury severity (*P *< 0.05). Circulatory i-FABP concentrations at ER correlated positively with IL-6 and PCT levels at the first day (r^2 ^= 0.19; *P *< 0.01 and r^2 ^= 0.36; *P *< 0.001 respectively) and CRP concentrations at the second day after trauma (r^2 ^= 0.25; *P *< 0.01).

**Conclusions:**

This study reveals early presence of intestinal epithelial cell damage in trauma patients. The extent of intestinal damage is associated with the presence of shock and injury severity. Early intestinal damage precedes and is related to the subsequent developing inflammatory response.

## Introduction

Severe trauma and major surgery frequently result in the development of inflammatory complications, including systemic inflammatory response syndrome, sepsis, and organ failure. These conditions are associated with a poor clinical prognosis [1,2]. For many years, the gut has been an organ of interest in the initiation and perpetuation of the inflammatory response following trauma or surgery [3-6]. In a rodent model of hemorrhagic shock that resembles the clinical situation of severe blood loss-induced splanchnic hypoperfusion, intestinal cell damage developed within one hour after shock induction [7]. Enterocyte damage following shock was paralleled by disruption of tight junction complexes and subsequent failure of the gut barrier. This resulted in translocation of luminal bacteria and toxins into the gut wall, which has been associated with the development of the inflammatory response [8-12]. Moreover, intracellular proteins that are released by damaged cells may contribute to the unfolding systemic inflammatory response by acting as damage-associated molecular patterns [13-15].

Although various animal studies indicate a role for gut integrity loss in the development of excessive inflammation following trauma, it remains to be clarified whether intestinal damage is present early after trauma in humans [16]. Some reports indicate that gut permeability as measured by sugar absorption tests is increased within 48 hours after trauma, which suggests that the intestine is compromised [17,18]. However, it is not resolved whether this is the cause or the consequence of systemic inflammation. Data on the state of the gut early after trauma are absent because the value of standard permeability tests is limited in the first hours [19].

This study aimed to clarify the early presence of enterocyte damage following trauma. To this end, on arrival at the emergency room (ER) circulating intestinal fatty acid binding protein (i-FABP), a specific biomarker for damage of differentiated enterocytes, was measured [20-24]. A second aim of this study was to gain insight into the factors that influence the development of intestinal cell damage following multiple traumas, such as presence of shock and injury severity. In addition, the relation between intestinal cell damage and the inflammatory response to trauma was explored.

## Materials and methods

### Patient selection

This prospective observational cohort study was approved by the Ethics Committee of J.W. Goethe University, performed in accordance with the Declaration of Helsinki and reported following the STrengthening the Reporting of OBservational studies in Epidemiology (STROBE) guidelines [25]. Informed consent was obtained by all patients or their relatives. Between April 2006 and December 2007, all trauma patients between 18 and 65 years were included at admittance to the ER. Exclusion criteria were burns, acute myocardial infarction, chronic inflammatory diseases, and lethal injury, resulting in a cohort of 96 patients.

### Assessment of shock and injury severity

Upon arrival at the ER, vital parameters of all patients were recorded. The shock index (SI) was calculated as a ratio between the first heart rate and systolic blood pressure registered. A normal SI was defined as a ratio of 0.7 or less [26]. Next, each injury was assigned an abbreviated injury scale (AIS) score ranging from 0 to 5. Each AIS score was allocated to one of six body regions (head/neck, face, chest, abdomen, extremities, and external) [27]. Of each body region, the highest AIS score was used. The injury severity score (ISS) was determined by squaring and adding together the scores of the three most severely injured body regions [28].

### Blood processing and analysis

Blood was withdrawn on arrival at the ER and daily during the patient's stay in the J.W. Goethe University Hospital until the second day after trauma. Samples were collected in pre-chilled EDTA vacuum tubes (BD vacutainer, Becton Dickinson Diagnostics, Aalst, Belgium) and kept on ice. Blood was centrifuged at 2000 g for 15 minutes at 4°C. The supernatant was stored at -80°C until batch sample analysis. Blinded specimens (n = 7) from trauma patients were used for duplicate measurement of i-FABP levels. i-FABP was determined using ELISA (kindly provided by Hycult Biotechnology, Uden, the Netherlands). i-FABP levels were also determined in 57 healthy volunteers between 18 and 65 years. For statistical analyses, the detection limit for i-FABP of 41 pg/mL was adjusted to samples in which i-FABP was not detectable (ER: 2 samples, day 1: 7 samples, day 2: 17 samples; and control: 20 samples). In the first 68 trauma patients, sufficient plasma was stored to study inflammatory parameters. Plasma concentrations of IL-6 were measured by ELISA (Diaclone, Hoelzel Diagnostica, Cologne, Germany) and C-reactive protein (CRP) using the Tina-quant CRP assay (Roche, Mannheim, Germany). Procalcitonin (PCT) levels were detected using a Kryptor-Assay (Brahms, Henningsdorf, Germany).

### Statistical analysis

First, the plasma i-FABP levels of all trauma patients on admittance and at days 1 and 2 were compared with healthy control values. Next, the relation between i-FABP values and the presence of shock and extent of injury severity were studied (general injury: ISS classified in five categories and abdominal injury: AIS). IL-6, PCT, and CRP levels in plasma were measured to analyze the inflammatory response in relation to early intestinal cell damage. A Kolmogorov-Smirnov test showed that plasma concentrations of i-FABP and inflammatory markers were not Gaussian distributed. Kruskal-Wallis test was used to analyze differences between groups with regard to the presence of shock, injury severity, and inflammatory markers. Mann-Whitney U test was used to compare separate groups. Data are expressed as median, 25th and 75th percentiles, and range in the figures and as median (range) in the text. A *P *value below 0.05 was considered to indicate statistical significance. After transformation of the data into natural logarithms, Spearman's correlation was used to assess the association between i-FABP and peak inflammatory parameters. Prism 4.0 for Windows (GraphPad Software Inc., San Diego, CA, USA) was used for computations.

## Results

### Intestinal cell damage is increased in trauma patients arriving at the emergency room

**Figure 1:**
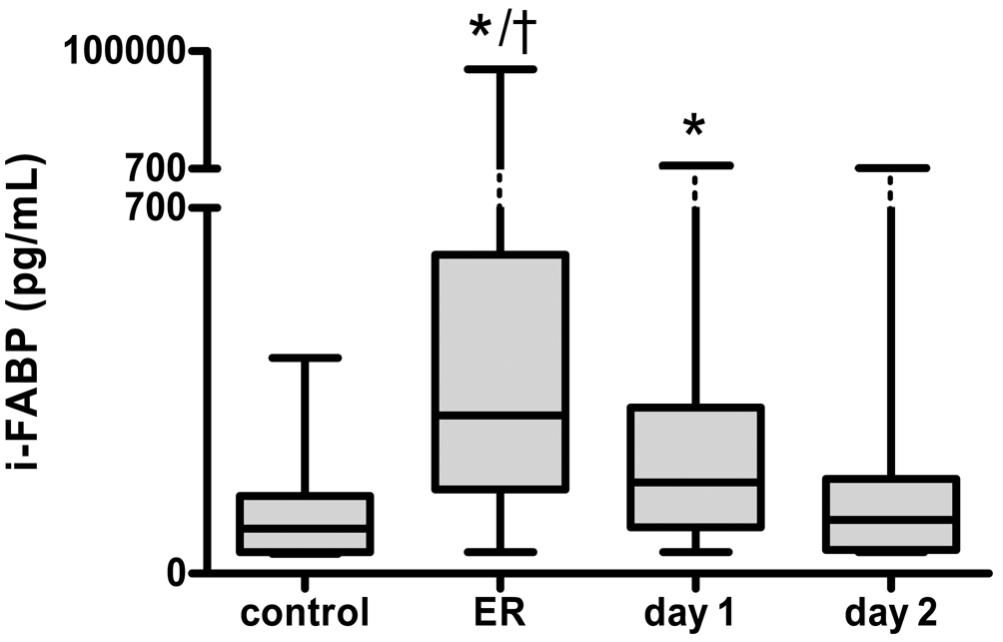
Intestinal cell damage increased rapidly following severe trauma. Plasma intestinal fatty acid binding protein (i-FABP) in trauma patients at the emergency room (ER) was significantly higher compared with samples collected at day 1 († *P *< 0.001), day 2 and healthy controls (both * *P *< 0.001). i-FABP concentrations at day 1 were elevated in comparison with day 2 and controls (both * *P *< 0.001).

The mean age of trauma patients (n = 96) was 40 years; 83% was male. Blood samples at admission to the ER were collected at a mean period of 85 minutes following trauma induction. Concentrations of i-FABP in trauma patients were significantly increased in comparison with healthy controls (303 (41 to 84,846) pg/mL vs. 87 (41 to 413) pg/mL; *P *< 0.001; Figure 1). i-FABP levels at ER were also elevated compared with levels at day 1 (174 (41 to 1805) pg/mL; *P *< 0.001) and day 2 (103 (41 to 1049) pg/mL; *P *< 0.001). At day 1, i-FABP concentrations were still increased compared with day 2 in control samples (both *P *< 0.001). i-FABP at day 2 was not significantly increased compared with control values (*P *= 0.21). Of all trauma patients at the ER, i-FABP levels of 89 patients (93% of all trauma patients) exceeded 87 pg/mL, which is the median of i-FABP plasma concentration in healthy controls.

### The extent of intestinal cell damage is related to presence of shock and injury severity

**Figure 2:**
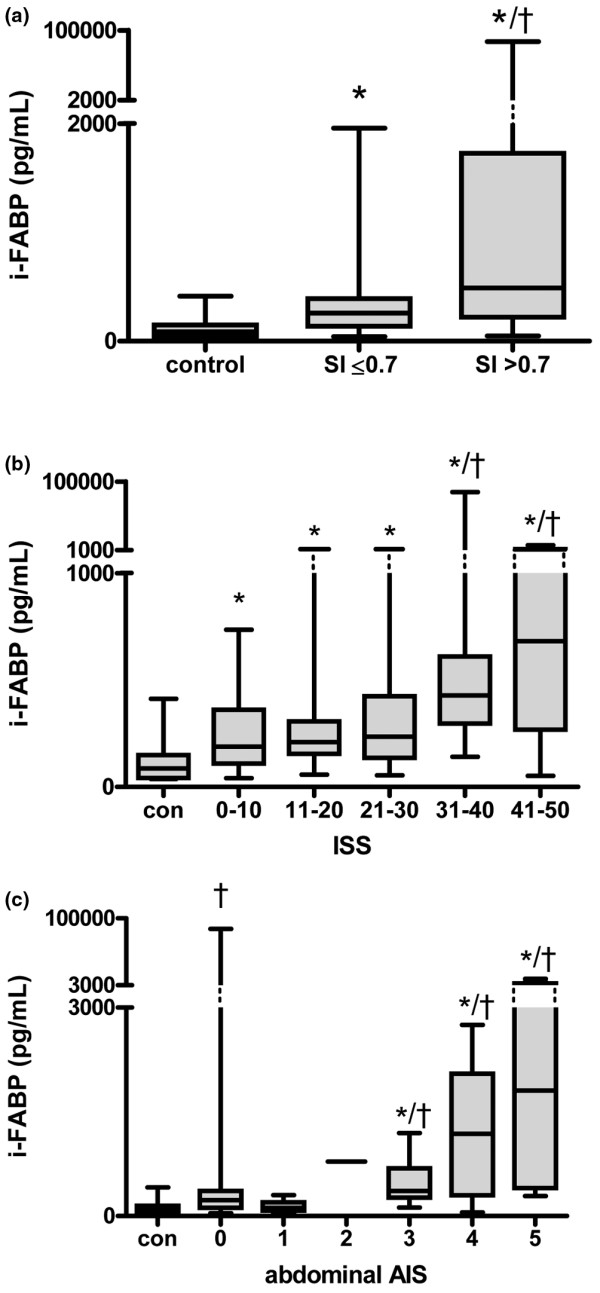
Early intestinal cell damage is related to presence of shock and the extent of injury severity. **(a) **Plasma intestinal fatty acid binding protein (i-FABP) concentrations were significantly increased in patients with an elevated shock index (SI > 0.7) compared with patients with a normal SI (= 0.7) († *P *< 0.01) or healthy controls (* *P *< 0.01). Also in trauma patients with a normal SI, i-FABP levels were higher in comparison to healthy controls (* *P *< 0.01). **(b) **i-FABP concentrations in patients with an injury severity score (ISS) of more than 30 were significantly elevated compared with ISS of 30 categories or less († *P *< 0.05). Intestinal cell damage in all ISS categories was increased compared with healthy controls (* *P *< 0.01). **(c) **i-FABP levels in patients with severe abdominal trauma (abbreviated injury scale (AIS) = 3) were significantly increased compared with patients without abdominal injury (AIS = 0; * *P *< 0.01) and healthy controls († *P *< 0.001). i-FABP levels in patients without abdominal trauma were significantly higher compared with healthy controls († *P *< 0.001).

To investigate the relation between hemodynamic stability and intestinal cell damage, i-FABP concentrations in trauma patients in shock were compared with patients without shock (SI > 0.7 vs. ≤ 0.7, respectively). On admittance to the ER, the SI was increased in 42% of the patients. Plasma i-FABP concentrations were significantly higher in patients with an elevated SI in comparison with patients with a normal SI (455 (41 to 84,242) pg/mL vs. 259 (41 to 1957) pg/mL; *P *< 0.01) or healthy controls (*P *< 0.01, Figure 2a). Also in trauma patients with a SI in the normal range, i-FABP levels were elevated in comparison to healthy controls (*P *< 0.01).

On admittance, the ISS of all patients was calculated and categorized. All ISS categories comprised 12 patients or more. i-FABP levels in patients with high ISS scores (ISS 31 to 40 and 41 to 50) were significantly increased compared with ISS 0 to 10, 11 to 20, and 21 to 30 categories (428 (142 to 84,846) pg/mL and 682 (52 to 8206) pg/mL vs. 189 (41 to 735) pg/mL, 210 (58 to 1860) pg/mL and 235 (54 to 1957) pg/mL, each *P *< 0.05; Figure 2b). In all ISS categories, intestinal cell damage was increased compared with healthy controls (*P *< 0.01).

Next, the severity of local abdominal trauma was assessed using the AIS scores of the abdomen. Scores of 0 (no abdominal injury), 3, and 4 (serious and severe abdominal injury) occurred most frequently (n = 48, 21, and 14 patients, respectively), whereas scores of 1, 2, and 5 were assigned less often (n = 8, 1, and 4 patients, respectively). As the abdominal AIS score of 2 was assigned only once, the i-FABP concentration detected in this patient (783 pg/mL) was not used for statistical evaluation. Taken together, at the ER abdominal trauma was diagnosed in 50% of the patients. i-FABP levels were significantly increased in patients with serious, severe, and critical abdominal injury (AIS 3: 364 (122 to 1194) pg/mL, AIS 4: 1185 (52 to 2753) pg/mL and AIS 5: 1806 (287 to 8206) pg/mL) compared with patients without abdominal injury (AIS 0: 231 (41 to 84,846) pg/mL; all *P *< 0.01) and healthy controls (all *P *< 0.001; Figure 2c). Interestingly, also i-FABP concentrations in patients without abdominal trauma were significantly elevated compared with healthy controls (*P *< 0.001).

### Remarkably elevated i-FABP values at admittance indicate abdominal emergencies

**Figure 3:**
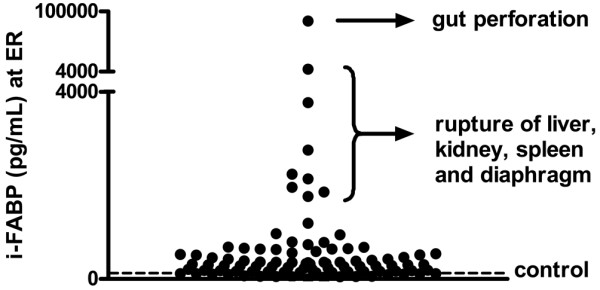
Strongly elevated i-FABP levels at ER indicate major abdominal trauma requiring immediate surgery. The highest 10% of intestinal fatty acid binding protein (i-FABP) values at emergency room (ER) were found in patients with severe abdominal trauma requiring acute intervention, such as rupture of the intestine, diaphragm, kidney, liver, or spleen.

In a few patients extremely elevated i-FABP levels were measured, far exceeding the values of other patients (Figure 3). Examination of the medical records revealed that the highest 10% of i-FABP values at ER belonged to patients with severe abdominal trauma that required acute surgical intervention, such as ruptures of the diaphragm, liver, and spleen. The highest i-FABP concentration (84,846 pg/mL) was measured in a patient assigned an AIS score of 0 at ER admission who was diagnosed at day 2 with intestinal perforation. In this patient, i-FABP concentrations at day 1 and 2 were 1181 pg/mL and 175 pg/mL, respectively.

### Intestinal mucosal cell damage correlates with the subsequent inflammatory response

**Figure 4:**
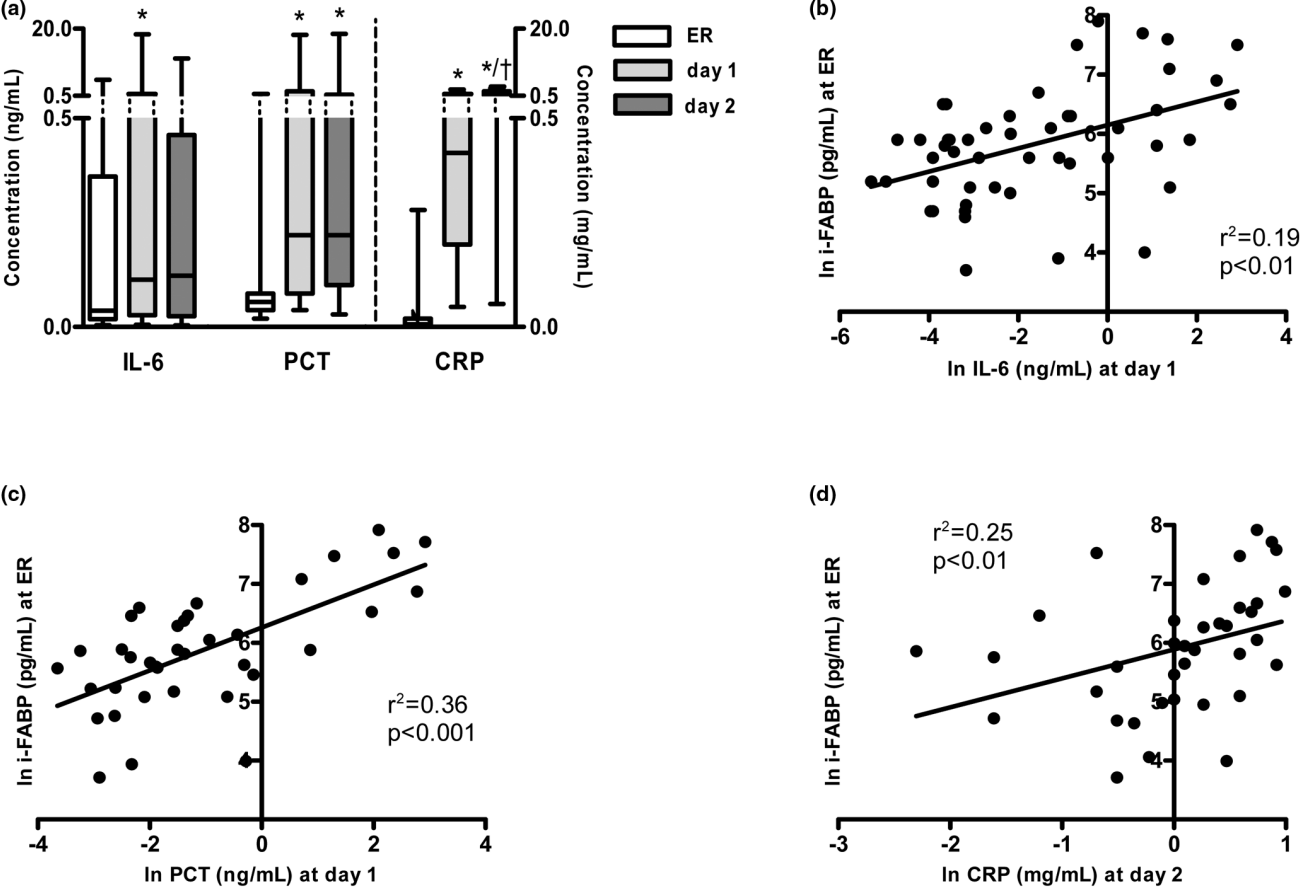
Intestinal mucosal cell damage after trauma correlates positively with the inflammatory response. **(a) **Peak concentrations of circulating IL-6 and procalcitonin (PCT) were reached at the first day after trauma, whereas highest levels of C-reactive protein (CRP) were measured at the second day (all parameters: * *P *< 0.05 vs. emergency room (ER); † *P *< 0.001 vs. day 1). **(b to d) **i-FABP concentrations at ER correlated positively with peak concentrations of IL-6 (r^2 ^= 0.19, *P *< 0.01), PCT (r^2 ^= 0.36, *P *< 0.001), and CRP (r^2 ^= 0.25, *P *< 0.01). All data are shown in natural logarithmic scale.

Circulating levels of IL-6, PCT, and CRP were measured on arrival at the ER and at the following days to explore the inflammatory response following trauma. Plasma IL-6 strongly increased at the first day (0.11 (0.01 to 18.35) ng/mL vs. ER: 0.04 (0.00 to 5.16) ng/mL, *P *< 0.05) and remained elevated at the second day (0.12 (0.00 to 11.37) ng/mL). Levels of PCT were barely detectable on presentation (0.06 (0.02 to 1.06) ng/mL), whereas elevated levels were measured at day 1 and 2 (0.22 (0.04 to 18.23) ng/mL and 0.22 (0.03 to 18.55) ng/mL, each *P *< 0.001 to ER). Consecutive measurements of acute phase protein CRP showed highest plasma values on the second day post-trauma (1.21 (0.06 to 2.72) mg/mL) compared with the first day (0.42 (0.05 to 1.57) mg/mL, *P *< 0.001) and to CRP concentrations on admittance (0.01 (0.00 to 0.28) mg/mL, *P *< 0.001; Figure 4a). Next we analyzed the relation between intestinal cell damage and the development of inflammation. Concentrations of i-FABP at admittance correlated positively with values of IL-6 (r^2 ^= 0.19, *P *< 0.01; Figure 4b) and PCT (r^2 ^= 0.36, *P *< 0.001, Figure 4c) on the first day after trauma. Furthermore, early i-FABP levels correlated with CRP in plasma at the second day (r^2 ^= 0.25, *P *< 0.01; Figure 4d).

## Discussion

Compromised intestinal integrity is considered to contribute to the inflammatory response to trauma [12]. This study sought to clarify the occurrence of intestinal damage and thus compromised integrity in the direct phase following severe trauma. Here, we showed presence of intestinal epithelial cell damage in a cohort of 96 trauma patients on arrival at the ER.

In the current study, evidence for intestinal cell damage after trauma was provided by increased plasma i-FABP levels. i-FABP is a small intracellular protein (14 kD) solely expressed in differentiated enterocytes of the small intestine and to a lower extent in the colon [20,22,24]. Following cell damage, i-FABP is released and readily detectable in circulation [22]. The fast clearance of FABP (T1/2 = 11 minutes) implies that the enhanced plasma i-FABP levels reflect ongoing intestinal damage in our study [29].

A strong increase of i-FABP was observed in trauma patients in shock. In the setting of shock, blood flow to the splanchnic region is hampered in favor of perfusion of vital organs such as the brain [30,31]. Therefore, the finding that i-FABP release is increased in shock is in line with studies that established splanchnic hypoperfusion as a major cause of i-FABP release. In a human model of gut ischemia and reperfusion, short-term ischemia induced a strong increase of plasma i-FABP, paralleled by histological damage of the epithelial layer and breakdown of intestinal barrier [22]. Moreover, elevated i-FABP levels were detected in settings of splanchnic hypoperfusion during non-abdominal surgery and critically illness [23,32]. In the present study shock was determined using the SI, which is considered more sensitive for shock than standard vital signs alone [26,33]. The observed mucosal epithelial damage in this patient cohort stresses the importance of rapid and adequate fluid administration after severe trauma [34].

An increase of intestinal cell damage was also present in patients with a normal SI, so the relation between intestinal damage and other trauma characteristics was explored. The ISS is a frequently used anatomical scoring system that correlates linearly with mortality, morbidity, hospital stay, and other measures of trauma severity [28]. In the current study, the extent of intestinal cell damage was found to be related to the ISS. It should be noted that the ISS is a composite of the scores of six body regions, including the abdomen. As abdominal trauma is a likely cause of intestinal cell damage, the relation between abdominal trauma and intestinal cell damage was then investigated. Half of the patients included in this study had trauma in the abdominal region, as determined using the AIS score. The highest i-FABP levels after trauma were detected in patients with severe abdominal trauma that required acute surgical intervention, such as rupture of the intestine, diaphragm, liver, and spleen. Further studies are needed to explore the sensitivity and specificity of i-FABP as an early marker for small intestinal organ damage following trauma. In addition to accepted diagnostic tools such as computed tomography, i-FABP assessment may help to detect abdominal emergencies in the early phase after trauma and support the decision to perform surgical intervention [24,34,35] (Relja and colleagues, unpublished data). In conclusion, the extent of intestinal cell damage is related to shock, ISS, and abdominal trauma.

In search for a potential role of the compromised gut in the development of inflammation following trauma, the relation between intestinal cell damage and the early inflammatory response was investigated. i-FABP levels on arrival at the ER correlated with concentrations at day 1 of IL-6, a potent cytokine in the early post-injury immune response that was identified as a useful predictor of complications as well as mortality [36,37]. Furthermore, i-FABP levels strongly correlated with day 1 plasma levels of PCT, an inflammation marker that is used to distinguish septic from non-septic patients [38]. In line, CRP concentrations at day 2 also correlated positively to early i-FABP values. Taken together, early intestinal cell damage clearly precedes and is related to the subsequent inflammatory response to severe trauma. Further studies are required to determine the causative involvement and predictive value of early enterocyte damage and gut barrier loss in the development of inflammatory complications.

The gut has long since been considered to play a role in the pathophysiology of complications following trauma [3-6]. Clarification of the role of the intestine in the development of excessive inflammation after trauma is not only interesting from an etiologic viewpoint, but may also contribute to the selection of patients for novel therapeutic strategies directed at preservation of intestinal integrity and attenuation of the inflammatory response [39].

## Conclusions

To the best of the authors' knowledge, this paper is the first to show that a significant proportion of trauma patients rapidly develops intestinal mucosal cell damage. The extent of intestinal damage is readily detectable in blood withdrawn on presentation at the ER. Circulatory concentrations of enterocyte damage marker i-FABP are related to the presence of shock and the extent of general injury as well as abdominal trauma, indicating that the level of intestinal cell damage is determined by both systemic and local factors. Moreover, early i-FABP values correlate positively with the inflammatory response that develops in the days following trauma. Further studies are needed to clarify the importance of early intestinal damage in the pathophysiologic response to trauma and its diagnostic and therapeutic implications.

## Key messages

• Intestinal mucosal cell damage develops early following trauma.

• The extent of intestinal damage is detectable in blood drawn on presentation at the ER.

• The presence of shock and the severity of local and overall injury are related to the extent of early intestinal cell damage.

• Early plasma values of intestinal epithelial cell damage marker i-FABP correlate positively with the subsequently developing inflammatory response.

## Abbreviations

AIS: abbreviated injury scale; CRP: C-reactive protein; ER: emergency room; i-FABP: intestinal fatty acid binding protein; IL: interleukin; ISS: injury severity score; PCT: procalcitonin; SI: shock index.

## Competing interests

WB is a shareholder of Hycult Biotechnology that provided the FABP assays.

## Authors' contributions

JdH coordinated the overall design of the study, analyzed the data, and drafted the manuscript. TL contributed to the design of the study, helped to interpret the data, and was involved in drafting the manuscript. JD helped to interpret the data and to draft the manuscript. BR collected the data and helped to analyze them. DH collected the data and helped to interpret the data. JG aided in defining the clinical context and revised the manuscript. IM conceived of the study, supervised the overall design, and revised the manuscript. WB conceived of the study, supervised the overall design, and helped to draft the manuscript. All authors read and approved the final manuscript.
